# Interaction of graphene nanoribbons with components of the blood vascular system

**DOI:** 10.4155/fso.15.17

**Published:** 2015-11-01

**Authors:** Sayan Mullick Chowdhury, Justin Fang, Balaji Sitharaman

**Affiliations:** 1 Department of Biomedical Engineering, Stony Brook University, Bioengineering Building, Room 115, Stony Brook, NY 11794–5281, USA

**Keywords:** endotheial toxicity, graphene, hematotoxicity, immunotoxicity, multiwalled carbon nanotubes, TEM

## Abstract

**Aim:**

The systemic administration of graphene nanoribbons for a variety of *in vivo* biomedical applications will result in their interaction with cellular and protein components of the circulatory system. The aim of this study was to assess the *in vitro* effects of graphene nanoribbons (O-GNR) noncovalently functionalized with PEG-DSPE (1, 2-distearoyl-*sn*-glycero-3-phosphoethanolamine-*N* [amino (polyethylene glycol)]) on some of the key hematological and vascular components of the circulatory system.

**Methods:**

Transmission electron microscopy was used to characterize the nanoparticles. ELISA-based assays, bright-field microscopy, transmission electron microscopy and colorimetric assays were used to assess toxicological effects.

**Results:**

Our findings taken together indicate that low concentrations of O-GNR-PEG-DSPE (<80 µg/ml) are relatively nontoxic to the hematological components, and could be employed for diagnostic and therapeutic applications especially for diseases of the circulatory system.

## Abstract (lay abstract)

Graphene nanoribbons are a class of carbon-based nanostructures derived from multiwalled carbon nanotubes that have been shown to have unique properties and high potential for drug-delivery applications in recent studies from our group. However, further development of this nanoparticle for biomedical applications will be possible only after its interactions with components of the circulatory system are suitably characterized. Toward that goal, this study is aimed at identifying potential toxicities of graphene nanoribbons in the circulatory system. Results from this study will give us indications about safe dosages and lay the foundation toward further animal studies.

**Figure 1.**
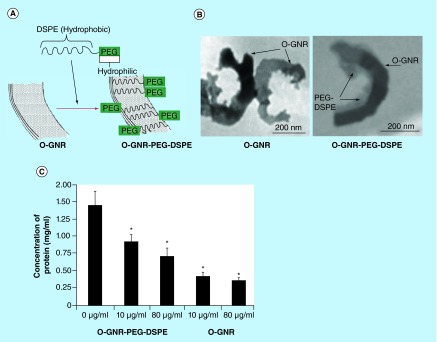
**Synthesis and protein binding of O-GNR-PEG-DSPE.** **(A)** Depiction of an O-GNR and O-GNR-PEG-DSPE (structures not to scale). **(B)** Representative TEM image of O-GNR and O-GNR-PEG-DSPE. **(C)** Concentration of human serum albumin in the supernatant of O-GNR and O-GNR-PEG-DSPE (at 10 and 80 µg/ml) treated protein solution centrifuged for 30 min at 3000 rpm. Concentration of the untreated but centrifuged control protein solution is provided as a reference. Data are presented as mean +SD (n = 4 per group). * = p < 0.05 between untreated control and particular treatment group. DSPE: 1,2-distearoyl-*sn*-glycero-3-phosphoethanolamine; O-GNR: Oxidized graphene nanoribbon; PEG: Poly-thylene glycol.

**Figure 2.**
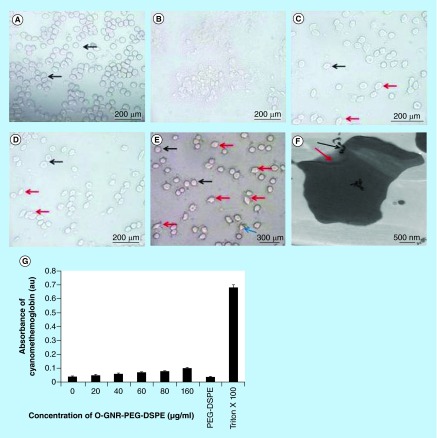
**Analysis of hemolytic potential of O-GNR-PEG-DSPE.** **(A)** Representative image of untreated control red blood cells. **(B)** Representative image showing hemolyzed cells treated with poly ethylene imine. **(C–E)** Representative images of blood cells treated with 20, 80 and 160 µg/ml GNR-PEG-DSPE for 3 h, respectively. In **(A–E)** black arrow represents a normal shaped RBC, red arrow represents abnormal shaped RBC and blue arrow represents lysed RBC. **(F)** Representative TEM image of a hemolyzed red blood cell treated with O-GNR-PEG-DSPE. Red arrow indicates lysed region and black arrow indicates the nanoparticles. **(G)** Absorbance at 540 nm obtained after conversion of the hemoglobin present in supernatant of red blood cells treated with the nanoribbons to cyanomethemoglobin. The supernatants were obtained by centrifuging red blood cell suspensions treated with GNR-PEG-DSPE formulations, PEG-DSPE or Triton X 100 for 3 h. Data are presented as mean +SD (n = 4 per group). * = p < 0.05 between untreated control and particular treatment group. O-GNR: Oxidized graphene nanoribbon; PEG-DSPE: 1, 2-distearoyl-*sn*-glycero-3-phosphoethanolamine-*N* (amino [polyethylene glycol]); RBC: Red blood cell.

**Figure 3.**
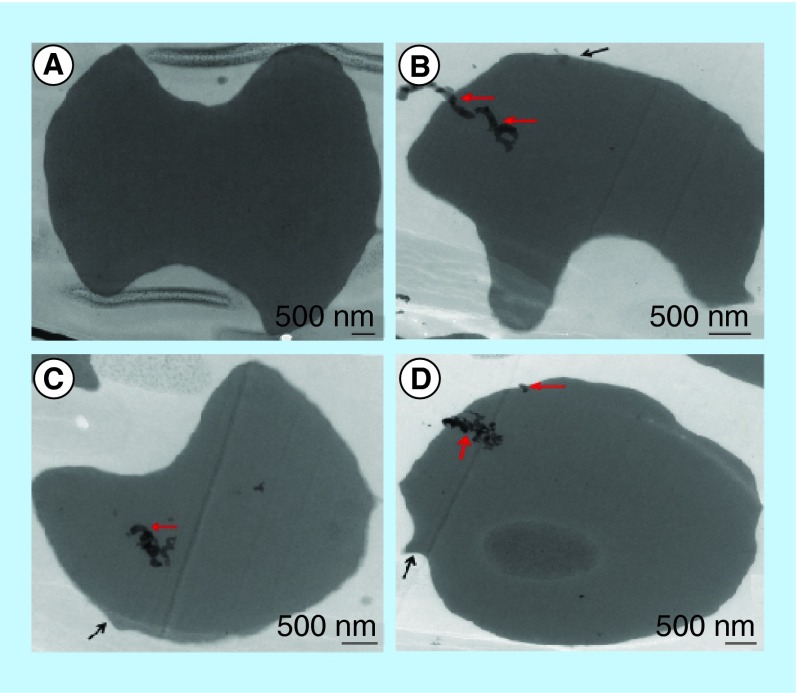
**Representative TEM images of red blood cells treated with 80 µg/ml O-GNR-PEG-DSPE for 3 h or left untreated.** **(A)** Representative TEM image of cross section of red blood cell not treated with O-GNR-PEG-DSPE. **(B)** Representative TEM image of cross section of a red blood cell treated with PEG-DSPE showing loss of concave shape on one side (black arrow). **(C)** Representative TEM image of cross section of a red blood cell treated with PEG-DSPE showing loss of concave shape on both sides. **(D)** Representative TEM image of cross section of a red blood cell treated with O-GNR-PEG-DSPE showing formation of a spherical cross section due to loss of structural integrity of the cells. O-GNR-PEG-DSPE particles are indicated with red arrows whereas protrusions/fragmentation from the membrane are indicated with black arrows. O-GNR-PEG-DSPE: Graphene nanoribbon, 1, 2-distearoyl-*sn*-glycero-3-phosphoethanolamine-*N* (amino [polyethylene glycol]).

**Figure 4.**
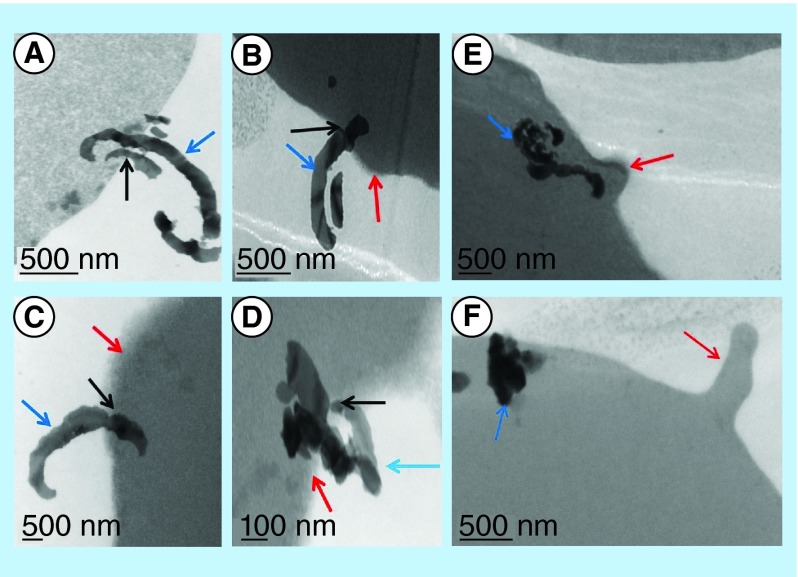
**Representative TEM images of red blood cells showing interaction of RBC membrane with 80 µg/ml O-GNR-PEG-DSPE.** **(A)** Representative TEM image of a red blood cell showing edges of O-GNR-PEG-DSPE in contact with RBC membrane. **(B&C)** Representative TEM images of red blood cells showing membrane disintegration/ruffling (red arrows) at the site of surface contact. **(D)** Representative TEM images of red blood cells showing membrane depression (red arrows) at the site physical contact with the nanoparticles. **(E&F)** Representative TEM images of red blood cells showing membrane protrusions/fragmentations near the sites where cell membrane comes in contact with O-GNR-PEG-DSPE. O-GNR-PEG-DSPE particles are indicated with blue arrows whereas protrusions/fragmentation from the membrane are indicated with red arrows. The site of contact of RBC membrane with O-GNR-PEG-DSPE particles is indicated with black arrows. O-GNR-PEG-DSPE: Graphene nanoribbon, 1, 2-distearoyl*-sn*-glycero-3-phosphoethanolamine-*N* (amino [polyethylene glycol]).

**Figure 5.**
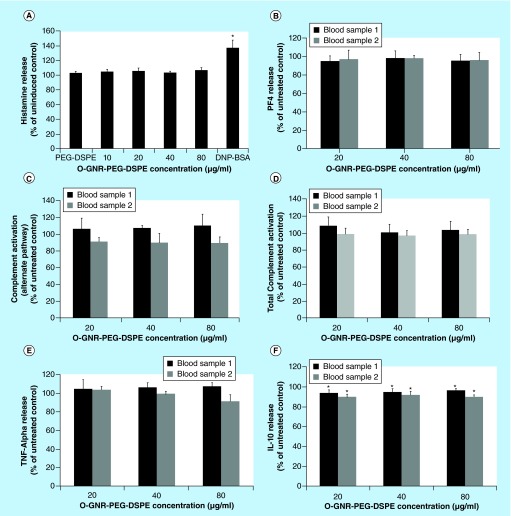
**Analysis of histamine release, complement activation, platelet activation and cytokine release.** **(A)** Histamine release from activated and induced RBL-2H3cells treated O-GNR-PEG-DSPE (0–80 µg/ml) formulations for 1 h. **(B)** Platelet activation assay presented in terms of PF_4_ production in whole human blood from two individuals incubated at 0–80 µg/ml O-GNR-PEG-DSPE concentrations for 1 h. **(C)** Total complement activation assay presented in terms of Sc5b-9 protein production in human whole blood from two individuals treated with various O-GNR-PEG-DSPE (0–80 µg/ml) concentrations for 1 h. **(D)** Alternate complement pathway activation in terms of Bb protein production in two human whole blood samples treated with various concentrations of O-GNR-PEG-DSPE (0–80 µg/ml) for 1 h. **(E)** Proinflammatory cytokine release assay presented in terms of TNF-α release in whole human whole blood from two individuals treated with various O-GNR-PEG-DSPE (0–80 µg/ml) concentrations for 1 h. **(F)** Anti-inflammatory cytokine release assay presented in terms of IL-10 release in whole human blood from two individuals treated with various O-GNR-PEG-DSPE(0–80 µg/ml) concentrations for 1 h. Data are presented as mean + SD (n = 4 per group). * = p < 0.05 between untreated control and particular treatment group. O-GNR-PEG-DSPE: Graphene nanoribbon, 1, 2-distearoyl-*sn*-glycero-3-phosphoethanolamine-*N* (amino [polyethylene glycol]).

**Figure 6.**
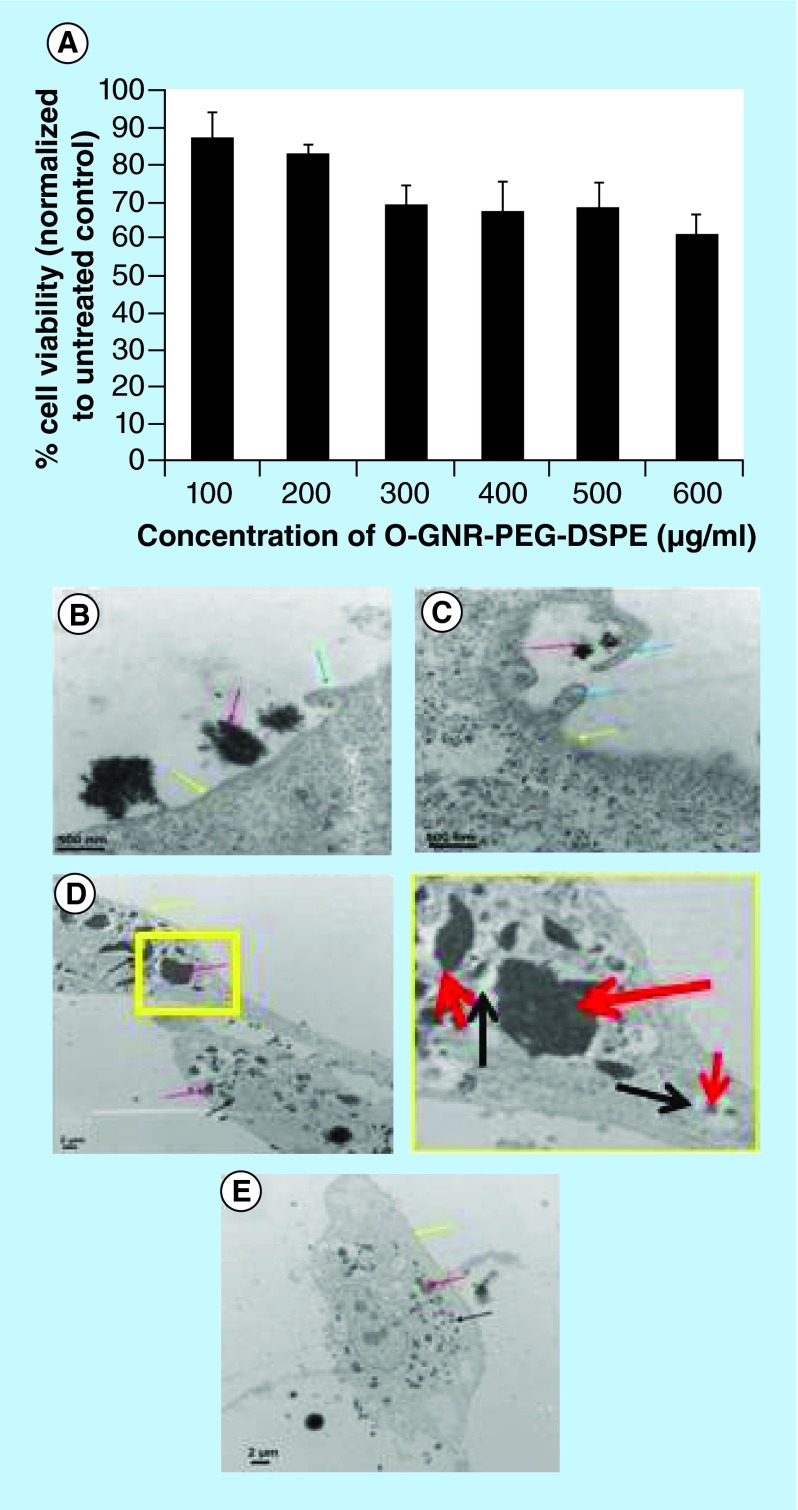
**Effect of O-GNR-PEG-DSPE on endothelial cells.** O-GNR-PEG-DSPE: Graphene nanoribbon, 1, 2-distearoyl-*sn*-glycero-3-phosphoethanolamine-*N* (amino [polyethylene glycol]).

Examination of the interactions between nanoparticles and cells and proteins present in tissues or circulating body fluids (blood, lymph, cerebrospinal fluid) is necessary while characterizing biological effects of nanoparticles [1,2]. Depending on the route and extent of exposure, nanoparticle-cell interactions can result in unwanted and detrimental effects [3]. Such effects are dependent on the size, shape, charge and surface characteristics of nanoparticles [3–5]. The type of interaction (i.e., direct cell interaction or indirect interaction after conjugation with other proteins) may also influence the observed effects [3,4]. Binding of proteins to nanoparticles may result in disruption of protein structure, unfolding of proteins or aggregation of proteins [1,3]. It may also lead to unwarranted activation of cellular receptors, resulting in a variety of responses, depending on the particular nanoparticle–protein interaction [3]. Thus, proper characterization of nanoparticle–protein and nanoparticle–cell interactions must be performed before any nanoparticle can be deemed suitable for biomedical use.

The use of nanoparticles for *in vivo* biomedical applications often involves their intravenous, intramuscular and intraperitoneal injection. This can result in interaction of the particles with different components of the circulatory system including blood proteins, clotting factors, blood cells and components of the immune and allergy response system. Thus, hematological toxicity of nanoparticles is a very critical component of its overall toxicological assessment. Hematological toxicity of nanoparticles has been extensively investigated in recent years. Reports suggest that manifestation of nanoparticle-induced hematological toxicity may vary and include increased or decreased cell counts (red and white blood cells), activation or inhibition of the immune response system, hemolysis, endothelial dysfunction and allergic responses. For example, gold nanoparticles [6], depending on their size, elicit an increase or decrease in red and blood cell count [6]. Iron oxide, Titanium dioxide, Silica and Carbon black nanoparticles have been shown to induce inflammation and endothelial dysfunction [7–10]. Zinc oxide nanoparticles have been shown to activate immune response [11]. Polymeric nanoparticles have been shown to decrease histamine release [12]. Single walled carbon nanotube dispersions, depending on their aggregation state, can induce either vasoconstrictory or vasodilatory responses in arterioles and endothelial dysfunction in the arterioles [13].

Graphene-based nanoparticles have shown promise for therapeutic drug-delivery and imaging applications. Graphene (also known as graphene oxide or graphene nanoplatelets) synthesized from graphite using modified Hummer's method (also known as graphene nanoplatelets) has been extensively investigated *in vitro* and *in vivo* [14–16]. Studies have examined the *in vitro* cellular as well as hematological toxicity of this particular form of graphene [17,18]. We recently reported that dextran functionalized graphene nanoplatelets decrease histamine release from rat mast cells and shows 12–20% increase in complement activation at high concentrations (>7 mg/ml) [18]. However, graphene nanoplatelets, unlike single walled carbon nanotubes, did not cause endothelial dysfunction [13,18]. These and studies on other carbon nanoparticles such as fullerenes and metallofullerenes [19], indicate that structure, chemical composition (pristine, functionalized) of carbon nanoparticles play an important role in their cellular interactions and associated hematotoxicity. Thus, structurally different carbon nanoparticles should be examined individually to better understand their specific hematotoxic responses.

Graphene nanoribbons (O-GNR) synthesized by oxidative unzipping of multiwalled carbon nanotubes [20] have also recently shown promise for bioimaging and drug-delivery applications [16,21–23]. O-GNR are thin long ribbon-like sheets of graphene with a large aspect ratio (ratio of length: breadth can be >10) and thus, structurally different than graphene nanoplatelets which typically have irregular or disc-shaped structure with a lower aspect ratio. Morphologically, O-GNR edges are different from graphene nanoplatelets due to the difference in the starting material [20]. Additionally, apart from the structural differences, O-GNRs are more oxidized compared with graphene nanoplatelets [20,24–26]. Previous cytotoxicity studies of water dispersible O-GNR (coated by amphiphilic polymer (1, 2-distearoyl-*sn*-glycero-3-phosphoethanolamine-*N* [amino (polyethylene glycol)]) (PEG-DSPE)) on various cell lines and stem cells demonstrated that they exhibit a significantly different cellular uptake characteristics and cytotoxicity profile compared with other types of graphene nanoparticles including graphene nanoplatelets [24,27]. Knowledge of the *in vitro* hematotoxicity of O-GNR-PEG-DSPE will assist in identifying potentially safe dosages for biomedical applications. Thus, we report the effects of interaction of O-GNR-PEG-DSPE (coated by PEG-DSPE and henceforth called O-GNR-PEG-DSPE) with red blood cells (RBCs) and other cellular and protein components of blood vascular system.

## Materials & methods

### O-GNR synthesis

O-GNRs were synthesized from multiwalled carbon nanotubes (MWCNTs) (Sigma-Aldrich, Length = 2.5–20 µm, Diameter = 6–13 nm) and noncovalently functionalized with PEG-DSPE (5000 KDa, Sunbright) using previously reported methods [20,24]. Briefly, MWCNTs (300 mg) were suspended in 60 ml of concentrated sulphuric acid (H_2_SO_4_) for 2 h. Potassium permanganate (KMnO_4_, 1500 mg) was added, and the mixture was stirred for 70 min. The reaction was heated at 55–70°C in an oil bath for an additional 1 h until completion, cooled to room temperature and washed with dilute aqueous hydrochloric acid. Ethanol and ether were added for flocculation, and the product was isolated by centrifugation at 3000 rpm for 30 min. The sample was then dried overnight in a vacuum oven. Dried O-GNR samples were weighed, and dispersed in 2 ml of PEG-DSPE, or DI water to obtain the different concentrations. The dispersions were bath sonicated for 25 min (Ultrasonicator FS30H, Fischer Scientific, PA, USA) for 25 min followed by probe sonication for 180 s (2 s on and 1 s off cycle, 20% amplitude, Cole Parmer Ultrasonicator LPX 750) to ensure homogenous O-GNR suspensions. Freshly-prepared O-GNR-PEG-DSPE formulations were used for all studies. We chose a straight(linear) chain PEG since that has been shown to be highly efficient for coating nanoparticles with drug-delivery applications [28]. Furthermore, higher molecular weight PEG has been shown to inhibit cellular uptake of nanoparticles. Hence, we used a PEG of medium molecular weight (5000 KDa) [28].

### Transmission electron microscopy

Samples for transmission electron microscopy (TEM) were prepared by dispersing the O-GNR in 1:1 mixture of water/ethanol by bath sonication for 1 min followed by ultracentrifugation at 5000 rpm for 5 min. The supernatant was dropped onto on formvar coated copper grids. The grids were then viewed with a Tecnai Bio Twin G TEM (FEI, OR, USA), at 80 kV. Digital images were acquired using an XR-60 CCD digital camera system (AMT, MA, USA).

### Cell culture

RBL-2H3 rat mast cells and Human umbilical vein endothelial cells (HUVEC) were obtained from ATCC (Manassas, VA, USA). RBL-2H3 cells were grown in a minimum essential medium with sodium pyruvate, nonessential amino acids and supplemented with 15% fetal bovine serum (FBS). One percent penicillin-streptomycin was used as antibiotic. Human umbilical vein endothelial cells were grown in F-12K medium supplemented with 10% FBS, 100 µg/ml heparin and 30 µg/ml endothelial cell growth supplement. Both cell lines were incubated at 37°C in a humidified atmosphere of 5% CO_2_, and 95% air.

### Protein binding

Pierce BCA protein assay kit was used to draw a standard curve for different concentrations (0–2 mg/ml) of Human serum albumin (HSA). HSA (2 mg/ml) was incubated with 10 µg/ml and 80 µg/ml O-GNR (no PEG-DSPE coating) and 10 µg/ml and 80 µg/ml O-GNR-PEG-DSPE on a shaker for 1 h at 37°C. Following the incubation all four samples were centrifuged at 3000 rpm for 30 min to pellet the O-GNR and O-GNR-PEG-DSPE along with any bound protein. The supernatant was collected and the concentration of proteins in the supernatant from each sample was estimated using Pierce BCA protein assay kit (Thermo Scientific, MA, USA) and the standard curve. An Evolution 300 UV-Vis spectrophotometer (Thermo Scientific) was used for the spectrophotometric measurements.

### Blood cell hemolysis

#### Cell morphology analysis

One milliliter whole human blood collected from a nonsmoking male (obtained from BioChemed, VA, USA) was treated with 20 µg/ml, 80 µg/ml and 160 µg/ml O-GNR-PEG-DSPE formulation or left untreated for 3 h. The treated or untreated blood was centrifuged at 2500 rpm for 10 min to separate the blood cell components. Two hundred microliter of the blood cell component was poured into 2 ml of isotonic buffer, and 15 µl of the resultant solution was streaked and fixed on a microscopic slide for imaging. The prepared slides were viewed under a bright-field microscope (Axiolab Microscope, Carl Zeiss, NY, USA). Polyethyleneimine, a known hemolytic agent was used as positive control, and phosphate buffered saline treated normal blood was used as negative control.

#### Hemoglobin release analysis

Release of hemoglobin from ruptured or lysed RBCs on exposure to various concentrations of O-GNR-PEG-DSPE was assessed by a method developed by McNeil *et al.* [29]. Briefly 2 ml of whole blood (Biochemed) was centrifuged at 2500 rpm for 15 min, and RBCs were separated out. The RBCs obtained as a pellet was carefully resuspended in 5 ml of phosphate buffered saline. O-GNR-PEG-DSPE was added to the suspensions to reach concentrations of 20, 40, 60 and 80 µg/ml and incubated for 3 h. Following this step, the mixtures were centrifuged at 2500 rpm for 15 min and the supernatants were removed. Ferricyanide along with bicarbonate was added to the removed supernatant, and incubated for 5 min. The absorbance of the resultant mixture was measured at 540 nm using an Evolution 300 UV-VIS Spectrophotometer (Thermo Scientific, FL, USA). Cells treated with PEG-DSPE alone were utilized as a negative control and cells treated with a known hemolytic agent Triton X 100 (1%) for 60 min were used as positive control.

### Interaction with red blood cells

One milliliter human whole blood was collected from a nonsmoking male individual (obtained from Biochemed) and centrifuged at 2500 rpm for 10 min to separate RBCs and plasma. The separated RBCs were diluted in 10 ml of phosphate buffered saline. Two milliliter of the diluted red blood cells were incubated with 80 µg/ml of O-GNR-PEG-DSPE or left untreated (control) on a shaker for 3 h at 37°C. At the end of 3 h, cells were centrifuged at 2500 rpm for 5 min and fixed with 2.5% electron microscopy grade glutaraldehyde (Electron Microscopy Sciences) in 0.1 M PBS. After fixation, the RBCs were placed in 2% osmium tetroxide in 0.1 M PBS, dehydrated using ethanol washes and embedded in durcupan resin (Sigma-Aldrich, MO, USA). The fixed and dehydrated RBCs were blocked, cut into 80 nm ultrathin sections using an Ultracut E microtome (Reichert-Jung, Cambridge, UK), and put on formvar-coated copper grids. The sections were then viewed with a Tecnai Bio Twin G transmission electron microscope (FEI), at 80 kV. Digital images were acquired using an XR-60 CCD digital camera system (AMT).

### Histamine release from mast cells

For this assay RBL-2H3 cells (10^4^ cells per well in 48 well plates) were sensitized by pretreatment with anti-2,4 dinitrophenyl (anti-DNP) IgE antibody (0.5 mg/ml) for 1 h at 37°C following which the cells were treated with PEG-DSPE (1.2 mg/ml), O-GNR-PEG-DSPE (10, 20, 40 and 80 µg/ml) and DNP-BSA (for inducing histamine release) for 1 h. Quantification of histamine release from RBL-2H3 cells treated with PEG-DSPE, O-GNR-PEG-DSPE and DNP-BSA was done using a histamine-O-phthalaldehyde (OPT) reaction which generates a fluorescent product. This assay procedure has been reported previously in detail by our group [18]. Briefly, in the first part of the assay, any histamine released from O-GNR-PEG-DSPE activated RBL-2H3 cells was collected into an organic phase from cell media treated with 0.4 N HClO_4._ The histamine collected into the organic phase is then returned to aqueous phase. In the final step histamine in aqueous phase is conjugated with OPT to produce a complex with excitation at 360 nm and emission at 450 nm. The fluorescence intensity produced is directly proportional to the amount of histamine that has been released. The fluorescence of the histamine-OPT conjugate was assessed at 450 nm emission after excitation at 360 nm in a Cytofluor fluorescence multiwell plate reader (Series H4000 PerSeptive Biosystems, MA, USA).

### Platelet activation

Immunoclone PF_4_ (Platelet Factor 4) ELISA kit (American Diagnostic, Inc., CT, USA) was used to assess platelet activation in terms of PF_4_ levels in whole human blood after treatment with 20, 40 and 80 µg/ml O-GNR-PEG-DSPE for 1 h. One hour nanoribbon incubation was used in this and other following studies (complement activation, TNF-α and IL-10 release) in accordance with previous reports [30,31]. Briefly, 1 ml human whole blood collected from two nonsmoking male individuals (hereafter called blood sample 1 and blood sample 2) (obtained from Biochemed) was treated with the three concentrations of O-GNR-PEG-DSPE or left untreated (control). The plasma from these untreated and treated samples was collected after centrifugation of the whole blood samples at 2500 rpm for 30 min. Plasma samples collected were diluted five-times using sample diluents provided with the kit. 0.2 ml of each plasma sample was incubated for 1 h in anti-PF_4_ coated wells following which each well was washed five-times with 0.3 ml wash solution provided in the kit. Postwashing 0.2 ml of anti-PF4-Horse radish peroxidase (HRP) immunoconjugate was added to each well and incubated for 1 h. Following this incubation the wells were washed again (five-times, using 0.3 ml wash solution each time). 0.2 ml of TMB substrate/peroxidase substrate (3, 3′, 5, 5′-Tetramethylbenzidine) was then added to the wells for 5 min at room temperature and 50 µl of 0.45 M H_2_SO_4_ was added, and incubated for 10 min to terminate the reaction. Absorbance of each well was measured at 450 nm using a microwell plate reader (ELx 800, BIOTEK, VT, USA).

### Activation of complement proteins

Microvue SC5b-9 and Bb plus ELISA kits (Quidel Corporation, CA, USA) were used to assess total complement activation and complement activation through the alternate pathway in whole human blood after treatment with 20, 40 and 80 µg/ml O-GNR-PEG-DSPE for 1 h. Briefly, 1 ml human whole blood collected from two nonsmoking male individuals (obtained from Biochemed) was treated with the three concentrations of O-GNR-PEG-DSPE or left untreated (control). The plasma from these untreated and treated samples was collected after centrifugation of whole blood samples at 2500 rpm for 30 min. Plasma samples from blood sample 1 were diluted five-times and from blood sample 2 were diluted three-times using sample diluents provided with the kit. Wells with antihuman SC5b 9/Bb coated micro well strip were incubated with 0.1 ml of diluted treated and control samples for 1 h. Following this step, each well was washed five-times with 0.3 ml wash solution provided with the kit, and 0.05 ml of anti-SC5b9-HRP Immunoconjugate/Bb-HRP immunoconjugate was added to each well and incubated for 30 min. The wells were washed again (five-times, using 0.3 ml wash solution each time), and 0.1 ml of TMB substrate/peroxidase substrate (3, 3′, 5, 5′-Tetramethylbenzidine) was added to the wells for 15 min at room temperature and then 0.1 ml of 0.45 M H_2_SO_4_ was added, and incubated for 10 min to terminate the reaction. Absorbance of each well was measured at 450 nm using a microwell plate reader (ELx 800, BIOTEK).

### TNF-α & IL-10 release

Human TNF-α and IL-10 ELISA kits (Invitrogen, NY, USA) were used to assess cytokine release in terms of TNF-α and IL-10 release in whole human blood after treatment with 20, 40 and 80 µg/ml O-GNR-PEG-DSPE for 1 h. Briefly, 1 ml human whole blood, collected from two nonsmoking male individuals) (obtained from Biochemed), was treated with the three concentrations of O-GNR-PEG-DSPE or left untreated (control) for 1 h. The whole blood samples were then centrifuged at 2500 rpm for 30 min and the plasma was collected from each sample. Wells, coated with antihuman TNF-α/anti-IL-10 antibody, were prepared by adding 50 µl of incubation buffer provided with the kit; 50 µl of the plasma samples (from treated and control) were then transferred to the appropriate wells, and incubated at room temperature for 2 h. The wells were then aspirated and washed four-times with wash buffer provided with the kit (0.3 ml per well per wash). 0.1 ml of biotinylated anti-TNF-α/IL-10 was then pipetted into the wells, and mixed. The wells were then incubated at room temperature for 2 h. Next, the wells were aspirated and washed four-times with wash buffer provided with the kit. 0.1 ml of streptavidin-HRP working solution was added to the wells, and incubated at room temperature for 30 min. The wells are subsequently aspirated and washed four-times (0.3 ml per well per wash). 0.1 ml of stabilized chromogen solution provided with the kit was next added to each well, which was incubated at room temperature for 30 min in the dark. The wells were finally added with 0.1 ml of stop solution, and read using an Infinite M200 multiwell plate reader (Tecan Group, NC, USA) at 450 nm absorbance.

### Effect on endothelial cells

#### Cell viability using presto blue assay

Cell viability in terms of mitochondrial integrity, and overall cellular metabolism was measured by presto blue assay (Invitrogen). Human umbilical vein endothelial cells were plated at 6 × 10^3^ cells per well in 96 well plates, and incubated for 18 h. Before commencing with the assay, old media was replaced with 150 µl of fresh media in each well. 50 µl of O-GNR PEG-DSPE stock solutions at various concentrations were added to every well for a final treatment concentration of 100, 200, 300, 400, 500 and 600 µg/ml. The cells were incubated at 37°C for 24 h. After the 24 h time point, media was removed, and wells were rinsed twice with Dulbecco's phosphate buffer saline before adding 100 µl of fresh media, and 10 µl of Presto Blue reagent. The plates were again incubated for 2 h at 37°C. Fluorescence readings of the wells were recorded using a Spectra Max M3 multimode microplate reader (Molecular Devices, CA, USA) with excitation at 530 nm, and emission at 580 nm. Fluorescence reading for cells in the culture medium containing only PEG-DSPE was used for baseline correction. The cell viability in terms of % of control cells is expressed as the percentage of (F_test_ – F_blank_)/(F_control_ – F_blank_), where F_test_ is the fluorescence of the cells exposed to nanoribbon sample, F_control_ is the fluorescence of the unexposed control sample and F_blank_ is the fluorescence of the wells without any cells.

#### TEM of endothelial cells exposed to O-GNR-PEG-DSPE

Six well plates with surfaces covered with ACLAR^®^ film (Electron Microscopy Sciences, PA, USA) were plated with cells at a density of 6 × 10^5^ cells per plate, and exposed to 40 µg/ml O-GNR-PEG-DSPE for 5 min or 12 h. At the end of two time points, cells were fixed with 2.5% electron microscopy grade glutaraldehyde (Electron Microscopy Sciences) in 0.1 M PBS. After fixation, the films containing fixed cells were placed in 2% osmium tetroxide in 0.1 M PBS, dehydrated through graded ethanol washes and embedded in durcupan resin (Sigma-aldrich). Areas with high cell densities were blocked, cut into 80 nm ultrathin sections using an Ultracut E microtome (Reichert-Jung, Cambridge, UK), and placed on formvar-coated copper grids. The sections were then viewed with a Tecnai Bio Twin G transmission electron microscope (FEI), at 80 kV. Digital images were acquired using an XR-60 CCD digital camera system (AMT)

### Statistical analysis

All data are presented as mean ±standard deviation. Student ‘*t*’ test was used to analyze the differences among groups. One-way analysis of variance followed by Tukey Kramer *post hoc* analysis was used for multiple comparisons between groups. All statistical analyses were performed using a 95% confidence interval (p < 0.05). n = 1 represents an average of three experiments done in parallel on the same day.

## Results characterization

### Transmission electron microscopy

Figure 1A shows depictions of O-GNR and O-GNR-DSPE. Figure 1B displays a representative TEM image of an O-GNR noncovalently functionalized with PEG-DSPE. TEM analysis indicated that the O-GNRs have multilayered structures with few defects mainly at the edges. The length of the O-GNR-PEG-DSPEs varied from 500 to 1500 nm and the width varied from 25 to 125nm (n = 15 particles). We have also previously exhaustively characterized the physiochemical properties of O-GNR PEG-DSPE [24–26]. Table 1 summarizes the salient physicochemical properties of O-GNR PEG-DSPE from those studies.

### Protein binding

Figure 1C shows the concentration of 2 mg/ml HSA solution remaining in the supernatant after incubation with O-GNRs with and without the PEG-DSPE coating. Two different concentrations (10 and 80 µg/ml) of O-GNRs with and without the PEG-DSPE coating were used. Untreated protein solution incubated for the same time as the treated solution showed a protein concentration of approximately 1.36 mg/ml. Concentration of the HSA in the supernatant after incubation with 10 and 80 µg/ml O-GNR (without PEG-DSPE coating) dispersions was 0.45 and 0.41 mg/ml (˜67 and ˜70% lower than untreated control protein solution), respectively. Concentration of the HSA in the supernatant after incubation with 10 and 80 µg/ml O-GNR (with PEG-DSPE coating) dispersions was 0.89 and 0.71 mg/ml (i.e., ˜33 and ˜50% lower than untreated control protein solution), respectively. In general, the results indicated a statistically significant increase in the concentrations of HSA in the supernatant solution of O-GNRs coated with PEG-DSPE compared with uncoated O-GNR's. Although 2 mg/ml is not a physiologically relevant range for HSA the BCA protein detection kit (as with moist protein detection kits) which we used for this assay allows detection up to 2 mg/ml for their standard curve. As such, we were limited by that for this assay. However, even at 2 mg/ml we get an indication that PEG-DSPE coating does decrease protein binding to O-GNR's.

### Blood cell hemolysis

Figure 2 qualitatively and quantitatively characterizes the extent of hemolysis of the RBCs after treatment with O-GNR-PEG-DSPEs using bright field optical microscopy and total hemoglobin release concentration. Figure 2A–E is representative bright field optical microscopy images of untreated RBCs or RBCs treated with different concentrations (0–160 µg/ml) of the nanoribbons for 3 h. Figure 2A shows the untreated control RBCs (black arrows) with a round morphology. Figure 2B shows the lysed control RBCs (lysed with polyethyleneimine) with an elongated morphology. Figure 2C shows morphologies of RBCs treated with 20 µg/ml O-GNR-PEG-DSPE. Most RBCs are structurally unaltered (black arrows). Few altered (red arrows) (compared with 2A) RBCs were noted (˜1% of total RBCs). Figure 2D shows morphologies of RBCs treated with 80 µg/ml O-GNR-PEG-DSPE. The figure shows more structurally altered RBCs (red arrows) compared with those treated with 20 µg/ml O-GNR-PEG-DSPE. However, no lysed cells were observed for both concentrations. Figure 2E shows RBCs treated with 160 µg/ml O-GNR-PEG-DSPE. The highest number of RBCs with changes in cell morphology was noted at this treatment condition (˜4% of total RBCs, red arrows). Very few lysed cells were noted at this concentration (blue arrow).

Figure 2G shows the amount of hemoglobin released from RBCs treated at various concentrations (0–160 µg/ml, for 3 h) of O-GNR-PEG-DSPEs. The hemoglobin was quantified by measuring the absorbance (at 540 nm) of a colored product (cyanomethemoglobin) formed by reacting the released hemoglobin with ferricyanide (in presence of bicarbonate). A very small increase in absorbance of cyanomethemoglobin (from ˜0.048 to ˜0.1 with increase in O-GNR-PEG-DSPE treatment concentration from 20 to 160 µg/ml) was detected implying very little hemoglobin was released in the supernatants. In comparison, lysed RBCs (using Triton X 100) showed a large increase in cyanomethemoglobin absorbance (˜0.7 A.U.) implying large amounts of hemoglobin were released in the supernatant.

### Interaction with red blood cells

RBCs with changes in their morphology (observed in Figure 2) were further qualitatively evaluated through histological analysis using TEM. Use of TEM for studying alterations in RBC shape is a well-documented, validated and widely used method reported in many previous studies [32–34]. Figure 3 shows representative cross-sectional TEM images of an untreated control RBC (Figure 3A), and a RBC (Figure 3B–D) after incubation with 80 µg/ml O-GNR-PEG-DSPE for 3 h. Figure 3B shows a RBC in the presence of O-GNR-PEG-DSPEs (red arrows). The RBC shows more prominent loss of its concave shape on the side in contact with the nanoribbons (black arrow). Figure 3C shows a RBC with O-GNR-PEG-DSPE aggregates (red arrows) on its surface, and complete loss of concave shape of this RBC on one side (black arrow) and partial loss on the other side. Figure 3D shows a RBC with O-GNR-PEG-DSPE aggregates on its membrane (red arrows), and complete loss of its biconcave shape. The shape of the RBC is circular; not characteristic of normal RBCs. Also visible are protrusions from the RBCs membrane (black arrow). Figure 3E shows a representative TEM image of one lysed RBC with a disrupted membrane (red arrows) and O-GNR-PEG-DSPE on the surface (black arrows).

Figure 4 shows representative TEM images of O-GNR-PEG-DSPEs on the surface of RBCs. The RBCs were treated with 80 µg/ml O-GNR-PEG-DSPE for 3 h. Figure 4A shows direct physical contact between the edges of O-GNR-PEG-DSPEs and cell membrane (black arrows). Figure 4B&C also shows edges of O-GNR-PEG-DSPEs physically touching the red blood cell membrane (black arrows). Additionally, the area near the site of contact appears blurred (red arrows) possibly due to membrane disintegration and/or ruffling. Figure 4D shows multiple O-GNR-PEG-DSPEs in contact with the cell membrane (black arrows). A depression in the cell membrane at the site of interaction (red arrows) is noted in the same figure. Figure 4E&F shows protrusions from RBC membrane near the sites where it interacts with O-GNR-PEG-DSPE (red arrows).

### Effect on mast cells: histamine release

The release of histamine from activated (with anti-DNP IgE)) RBL-2H3 rat mast cells was measured by extraction of histamine from cell media followed by a histamine-OPT reaction that generates a fluorescent conjugate that is quantified. Figure 5A shows the histamine released from activated RBL-2H3 rat mast cells treated with three concentrations of O-GNR-PEG-DSPEs (20, 40 or 80 µg/ml, for 1 h) and DNP-BSA (inducer). Activated mast cells left untreated and treated with PEG-DSPE were used as controls. The results are expressed as a percentage of histamine released from the untreated mast cells. Cells treated with only DNP-BSA produced an approximately 40% increase in histamine release compared with the activated but uninduced cells. This value is similar to our previous studies using this particular assay [18]. In contrast, cells treated with O-GNR-PEG-DSPE (at 20, 40 and 80 µg/ml) or PEG-DSPE alone did not show statistically significant increase in histamine release.

### Effect on platelets: PF_4_ release

PF_4,_ a heparin binding and deactivating protein is the most abundant protein found in platelets. It is released into the plasma once platelets are activated. Hence PF_4_ concentration in plasma is an efficient marker for platelet activation in blood. Evaluation of platelet activation potential of O-GNR-PEG-DSPE is of paramount importance since platelet activation may lead to platelet aggregation and blood clotting. An ELISA assay that utilizes anti-PF_4_ antibody was performed to detect the amount of PF4 in blood. Figure 5B shows PF4 levels in two different samples of whole human blood after treatment with O-GNR-PEG-DSPEs at three concentrations (20, 40 and 80 µg/ml, for 1 h). The results are expressed as a percentage of the PF4 levels in blood that was not treated with the nanoribbons. No statistically significant changes in PF_4_ levels were observed in the two blood samples after treatment with O-GNR-PEG-DSPE at the three concentrations. Positive control samples provided with the kit showed an approximately 400% increase in PF_4_ indicating that the assay plate and reagents were working.

### Interaction with complement proteins

Figure 5C shows the total complement activation presented in terms of SC5b9 protein levels in plasma, after treatment of whole human blood from two individuals with O-GNR-PEG-DSPEs at three concentrations (20, 40 and 80 µg/ml, for 1 h). The data are presented as percentage of SC5b-9 levels in untreated control blood. The SC5b-9 or terminal complement complex assay is based on the principle that all forms of complement activation (i.e., classical, lectin and alternate pathway) leads to formation of SC5b-9. Hence, it is considered an excellent marker for analysis of total complement activation. The assay utilized for detection of Sc5b-9 uses a monoclonal antibody that binds to the C-9 ring of the SC5b-9 protein. The results indicate that concentrations up to 80 µg/ml of O-GNR-PEG-DSPE do not significantly alter the levels of SC5b9 in both the blood specimens, compared with untreated control blood. Positive control samples provided with the kit showed an approximately 430% increase in SC5b9 indicating that the assay plate and reagents were working.

Figure 5D shows the alternate pathway activation presented in terms of Bb protein levels in plasma, after treatment of whole human blood from two individuals with O-GNR-PEG-DSPEs at three concentrations (20, 40 and 80 µg/ml, for 1 h). Bb protein is an activation product specific for activation of the alternate pathway. It is a small protein fragment produced due the cleavage of factor B by factor D. The assay kit employs a monoclonal antibody against Bb protein. The data are presented as percentage of Bb levels in untreated control blood. No statistically significant changes in Bb levels were observed in the two blood samples after treatment with O-GNR-PEG-DSPE at the three concentrations. Positive control samples provided with the kit showed an approximately 280% increase in SC5b9 indicating that the assay plate and reagents were working.

### Effect on macrophages & monocytes: TNF-α and IL-10 release

Figure 5E&F shows the amount of TNF-α and IL-10 released from the two whole human blood samples treated with 20, 40 or 80 µg/ml O-GNR-PEG-DSPEs for 1 h. TNF-α is a proinflammatory cytokine and IL-10 is an anti-inflammatory cytokine. These cytokines are released from cells of the innate immune system like macrophages and monocytes in response to external irritants, pathogens or foreign particles. Normally these cells maintain a balance between the secretion of pro- and anti-inflammatory cytokines. However, upon contact with a foreign body, these cells can increase or decrease the secretion of one or both kinds of cytokines; thereby promoting or inhibiting inflammation. Quantification of TNF-α was performed using an ELISA kit that employs an anti-TNF-α antibody. Results are expressed as percentage of control TNF-α levels in untreated blood. The results (Figure 5F) show no significant difference in the release of TNF-α between untreated or treated whole blood samples. Quantification of IL-10 was done using an ELISA kit with an anti-IL-10 antibody. Figure 5G shows the percentage of IL-10 released from whole human blood of two individuals treated with 20, 40 and 80 µg/ml O-GNR-PEG-DSPE. Results are expressed as percentage of control IL-10 levels in untreated blood. The figure shows a decrease in IL-10 release (by ˜5–7%) for blood sample 1 (˜7% decrease for 20 µg/ml, ˜6% decrease for 50 µg/ml and ˜5% decrease for 80 µg/ml) and (˜8–10%) for blood sample 2 (˜10% decrease for 20 µg/ml, ˜8% decrease for 50 µg/ml and ˜9% decrease for 80 µg/ml). Positive control samples provided with the kit showed an approximately 300% increase in TNF-α and IL-10 indicating that the assay plate and reagents were working.

### Endothelial cell viability using presto blue assay

Figure 6A shows the viability, evaluated using the presto blue assay, of HUVEC treated with different concentrations of O-GNR-PEG-DSPE (100–600 µg/ml for 24 h). The data are presented as percentage of untreated control cells. The presto blue assay reagent is a resazurin-based compound that is reduced from its nonfluorescent (resazurin) form to a highly fluorescent (resosurfin) form by enzymes in a living cell. Thus, more number of viable cells generate greater fluorescence which can be measured to quantify cell viability. The results show a concentration dependent decrease in cell viability. Treatment of HUVEC cells at the two lowest concentrations of O-GNR-PEG-DSPEs (100 and 200 µg/ml) resulted in a decrease in viability by 15 and 18%, respectively. Treatment of HUVEC cells with 300 and 400 µg/ml O-GNR-PEG-DSPE leads to an approximately 30 and 31% decrease of viability, respectively. Treatment of HUVEC cells at the two highest concentration of O-GNR-PEG-DSPEs (500 and 600 µg/ml) resulted in an approximately 32 and 40% decrease in cell viability, respectively.

### TEM of endothelial cells exposed to O-GNR-PEG-DSPE

Figure 6B&C and Figure 6D&E are representative TEM images of histological specimens of HUVEC cells treated with 40 µg/ml O-GNR-PEG-DSPE for 5 min and 12 h, respectively. We did a systematic study to identify the best concentration of O-GNR-PEG-DSPE for TEM images of HUVEC cells. The concentration 40 µg/ml was used since at this concentration was low enough to not interfere with the processing for TEM images while it was also high enough to show observable effects and interactions of the nanoparticle and the cells. Figure 6B shows O-GNR-PEG-DSPE aggregates (red arrow) near the cell membrane (yellow arrow). Membrane protrusions (blue arrow) from the cells are also noted. Figure 6C shows O-GNR-PEG-DSPE aggregates (red arrow) surrounded by membrane protrusions (cell membrane indicated with yellow arrows and protrusions indicated with blue arrows). Figure 6D&E shows O-GNR-PEG-DSPE aggregates (red arrows) within vesicles (black arrows) uptaken into cells. The yellow arrow points to the cell membrane. The aggregates are seen mostly in the periphery of the cell.

## Discussion

The overall goal of studies presented in this article was to investigate the interaction between O-GNR-PEG-DSPE and the cellular and protein components of the blood vascular system. The experimental design of this study was based on previous reports on possible hematological toxicity mechanisms associated with nanoparticles. For future preclinical small animal studies, based on our previous *in vitro* work, we anticipate the steady state blood concentration of O-GNR-PEG-DSPE to be 1–25 g/ml (˜0.048–1.2 mg/kg in rats, assuming an average weight of rats as 250 mg and a circulating blood volume of ˜12 ml). Previous studies have shown that, as bolus injected nanoparticles undergo dilution in blood through circulation the cellular and protein components of blood are subject to several times higher concentration of nanoparticles compared with the steady state concentration [13]. As such, we tested the effects of O-GNR-PEG-DSPE (up to 160 µg/ml in hemolysis study and 80 µg/ml in other studies) on components of the blood vascular system. Furthermore, blood vessels at the site of injection can be subject to even higher concentration of nanoparticles (˜12-times higher in rats assuming a circulating blood volume of 12 ml) compared with the steady state blood concentration [13]. As such, toxic effect of up to 600 µg/ml (24-times higher than the highest anticipated concentration) O-GNR-PEG-DSPE was evaluated on endothelial cells. Graphene nanoribbons, synthesized from MWCNT vary in dimensions depending on the size of the starting material (i.e., MWCNT) and the time of oxidation [20]. The values of lengths (500–1500 nm) and widths (25–125 nm) of the O-GNR-PEG-DSPEs are in agreement with literature values reported by us and others [20,24–27]. Even though O-GNRs are more dispersible in water compared with other types of graphene nanoparticles [20], noncovalent functionalization with PEG-DSPE (forming O-GNR-PEG-DSPE) (Figure 1A&B) improves their stability in aqueous suspensions [24]. Additionally, PEG-DSPE coating could significantly reduce to nonspecific absorption of blood proteins onto O-GNRs. Immediately after administration, blood proteins could form a ‘corona’ of proteins around hydrophobic nanoparticles [35]. This nonspecific protein absorption affects the biodistribution and uptake of nanoparticles into immune competent and phagocytotic cells [35]. Typically, to prevent nonspecific protein absorption (especially for charged nanoparticles which bind more proteins compared with neutral nanoparticles), nanoparticles are often covalently or noncovalent functionalized with polymers such as PEG. The noncovalent PEG-DSPE functionalization of O-GNRs showed a concentration dependent decrease in the absorption of albumin (the most abundant protein in blood) onto O-GNRs (Figure 1C). The functionalization substantially mitigated, but did not eliminate the absorption of albumin (Figure 1C). The untreated protein solution also showed decrease in protein concentration due to binding of some proteins to the walls of the centrifuge tubes after centrifugation.

*In vivo*, circulating O-GNR-PEG-DSPEs will come in contact with the RBCs (which are the most abundant cells in blood). RBCs are devoid of nucleus and are characterized by their biconcave shape [36]. Maintenance of the biconcave shape is essential for movement of RBCs through blood vessels and capillaries [36]. The RBC membrane is supported by a cytoskeleton made up of spectrin, actin and ancillary proteins (ankyrin, protein 4.1 etc.,) that support the biconcave shape [37]. Interaction of nanoparticles with RBCs has been shown to cause membrane penetration and deforms the RBCs as well as cytoskeletal disruption leading to hemolysis [38]. In two recent studies, pristine graphene nanoparticles and functionalized MWCNTs have been shown to induce hemolysis by disrupting the cell membrane [39,40]. We have previously shown that functionalization of graphene nanoplatelets with dextran can mitigate the observed hemolysis in graphene [18]. In case of O-GNR-PEG-DSPE exposure, bright field images of RBCs showed a concentration dependent increase in the number of deformed cells (Figure 2). A low number (in comparison with Triton X 100 treated cells) of deformed cells at all concentrations, and very few lysed cells at higher (160 µg/ml) concentrations were observed by bright-field optical microscopy. Figure 2A–E suggests that at concentrations <160 µg/ml, although some cells may change upon interfacing with O-GNR-PEG-DSPEs, the interactions do not lead to cell lysis. The insignificant increase in hemoglobin release from RBCs at all treatment concentrations with O-GNR-PEG-DSPE further corroborated this assessment.

Even though, only a few RBCs showed evidence of structural deformation, further in-depth analysis of the deformed RBCs performed using TEM (Figure 3) showed partial (Figure 3B&C) or complete (Figure 3D) loss of their biconcave structure. Upon losing their biconcave morphology, RBCs transform into spherocytes, in other words, spherical RBCs [41], considered to be abnormal cells and removed from circulation by the reticuloendothelial system [42]. In these deformed cells, the site of interaction between the RBC membrane and O-GNR-PEG-DSPE showed blurriness and depressions in the membrane (Figure 4) suggestive of a change in cytoskeletal organization in that region (Figure 4D). The membrane dynamics of RBCs are usually very tightly regulated and extensions from fragmentation of the membrane surface is not observed due to the rigid cytoskeletal structure [43]. The membrane extensions from RBCs near the site of their interface with O-GNR-PEG-DSPE (Figure 4E&F) suggest breakdown in cytoskeletal structure; characteristic of spherocyte formation [44]. Although, direct interaction of nanoribbons may be a probable cause, disruption of the cellular cytoskeletal architecture may take place by several other mechanisms such as aberrant receptor activation and reactive oxygen species generation and needs to be further investigated [45,46].

The probability of O-GNR-PEG-DSPEs interacting with other blood cells (white blood cells, platelets) and proteins (complement proteins) would be lower than RBCs as total volume of RBCs in blood is significantly higher compared with the other cells and proteins., However, the other blood cells modulate functioning of the immune and allergen response system. Thus, the effects of O-GNR-PEG-DSPEs on other blood cells and proteins were also examined. Histamine is an strong indicate of allergen response [47]. Histamine, a bioactive amine, is stored as granules in circulating basophils and mast cells that need to be activated (through immunoglobulin E) and induced by an allergen to degranulate and secrete the stored histamine from these cells [48]. Once released, it can interact with receptors on cells of different tissues to produce different physiological and pathological effects [49]. Titanium dioxide nanoparticles have been shown to induce and activate mast cells to release histamine release leading to an allergen response [50]. Recently, exposure of dextran coated graphene oxide nanoplatelets to both mast cells and human blood resulted in decreased histamine release compared with unexposed controls at high concentrations (>3 mg/ml) [18]. Interestingly, our results (Figure 5A) show that up to 80 µg/ml O-GNR-PEG-DPE does not induce histamine release from rat mast cells. This result suggests that graphene structures with different morphologies can elicit dissimilar allergic response upon exposure to the same cells or tissues.

Platelets in blood circulate in their inactive form and are activated through breakage or disruption in the endothelium of blood vessels [51]. Activation of platelets ultimately leads to the clotting of blood at the site of endothelium breakage [51]. Foreign particles in blood may interact directly with platelets and induce their activation cascade [52]. Clots formed without breakage in endothelium might result in blockage of blood flow through the vessel which could be fatal [53]. Carbon nanoparticles like single and multiwalled carbon nanotubes (tested up to 160 µg/ml) and graphene nanoplatelets (tested up to 2 µg/ml) have been previously shown to activate blood platelets leading to platelet aggregation and formation on clots [39,54,55]. Exposure of O-GNR-PEG-DSPE concentrations (up to 80 µg/ml for 1 h) did not significantly alter the level of activation of platelets in whole human blood (Figure 5B). This result indicates that interaction of O-GNR-PEG-DSPEs under these conditions may not induce the activation cascade in platelets.

Activation of the complement proteins in blood is an important biocompatibility test for biomaterials [56]. Hypersensitivity reactions are common in case of unwanted activation of the complement system. The complement system comprises a set of proteins that are involved in the immunological response to foreign bodies or antigens [56]. The three main pathways involved in complement activation are the classical pathway (antigen antibody complexes are formed that lead to a cascade of protein cleavage reactions resulting in the activation of the complement system); alternate pathway (does not involve formation of antigen antibody complex but involves spontaneous activation of the protein cleavage reactions in response to the antigen) and the lectin pathway (initiated by mannose binding lectin binding to antigens) [56]. Biomaterials once injected *in vivo* usually trigger the alternate or lectin pathway but do not usually affect the classical pathway [56]. A variety of nanoparticles have been reported to activate the complement system through different mechanisms depending on their structure. For example, pegylated single walled carbon nanotubes have been reported to activate the complement system (treated up to 80 µg/ml for 10 min) through the lectin pathway [57]. Pegylated Doxil^®^ liposomal nanoparticles have been reported to activate the alternate pathway [58]. Dextran functionalized graphene nanoplatelets showed small increase in total complement activation (12–20%). O-GNR-PEG-DSPEs, under the tested conditions (20–80 µg/ml for 1 h) do not induce activation of any form of the complement system in the two blood samples tested (Figure 5C&D). The results indicate that under these conditions interaction of the O-GNR-PEG-DSPE particles with the complement proteins does not result in induction of the protein cleavage cascade.

Macrophages and monocytes are part of the immune system that phagocytose as well as regulate the release of proinflammatory or anti-inflammatory cytokines in response to a pathogen or irritant [59]. Normally, a balance between pro and anti-inflammatory cytokines is maintained in the body [60]. On encountering an antigen, the balance is shifted either toward proinflammatory or anti-inflammatory cytokines depending on the type of antigen encountered with a simultaneous decrease in the other kind of cytokine [60]. Thus, a change in the equilibrium would mean the macrophages or monocytes have been activated to release these cytokines. Previous studies with nanoparticles have shown that depending on their type, nanoparticles can potentially have both proinflammatory [61,62] and anti-inflammatory effects [63]. Furthermore, depending on the size and the method of production nanoparticles of the same composition may produce drastically opposite effects on release of inflammation associated cytokines [63,64]. Thus, it is essential to evaluate the effects of any nanoparticle on pro- and anti-inflammatory cytokine release. A recent report by our group has shown that dextran functionalized graphene nanoplatelets do not induce significant changes in pro- or anti-inflammatory cytokine release [18]. O-GNR-PEG-DSPEs did not increase TNF-α levels (a proinflammatory cytokine) in whole blood (Figure 5E). However, a small decrease in IL-10 (an anti-inflammatory cytokine) release (by 5–10%) was observed (Figure 5F). The concentration of IL-10 (˜1 pg/ml) for both control and treated blood samples was within the normal limits (<3 pg/ml). Thus, under the tested conditions O-GNR-PEG-DSPE does not cause potentially toxic changes in release of inflammation related cytokines.

Circulating O-GNR-PEG-DSPEs could interact with endothelial cells lining the blood or lymphatic vessels. Once administered, the endothelial cells at the site of injection would be subject to several times higher concentration of O-GNR-PEG-DSPE compared with the steady state concentration in blood achieved after several passes through the circulatory system [13,18]. Previous studies have shown that nanoparticles can interact with the endothelial lining of blood vessels to cause endothelial dysfunction. This effect, whose mechanism is nanoparticle specific, has been observed in a variety of nanoparticles including iron oxide nanoparticles, carbon black nanoparticles and silica nanoparticles [7,10,65]. A recent study by our group has also shown that single walled carbon nanotubes can induce a dose dependent endothelial dysfunction [13]. However, high concentrations of dextran coated graphene nanoplatelets did not elicit a similar response [18]. Thus, it is essential to investigate the effects of O-GNR-PEG-DSPEs on endothelial cells as a first step in examining its propensity to cause endothelial dysfunction. To this end, Human umbilical vein endothelial cells were treated with O-GNR-PEG-DSPEs. Direct exposure of these nanoparticles does not mimic the actual situation where O-GNR-PEG-DSPE will flow (along with the blood) over these cells. However, it would provide insights into endothelial cell O-GNR-PEG-DSPE interactions, and the concentrations to that could adversely affect these cells. We observed a dose dependent decrease in cell viability (Figure 6A). The lowest concentration (100 µg/ml) showed approximately 15% decrease in cell viability compared with untreated controls. Increase in treatment concentration to 500 µg/ml showed approximately 25% decrease in cell viability. Further, the CD_50_ concentrations of O-GNR-PEG-DSPE were not reached even at the highest treatment concentration of 600 µg/ml (Figure 6A). Histological evaluation of the cells by TEM suggested that the endothelial cells generate cell membrane protrusions that engulf the O-GNR-PEG-DSPE aggregates and take in large amounts into vesicular structures (Figure 6B–E). This phenomenon of O-GNR-PEG-DSPE accumulation in vesicular structures is similar to our observations in previous studies [22–24]. However, the intracellular accumulation of O-GNR-PEG-DSPE observed in this study was significantly higher than that observed in the previous studies done in HeLa cells [22–24]. The dose dependent cytotoxicity observed may be due to the significant uptake of O-GNR-PEG-DSPE that could affect the regular functioning of cell organelles; previously observed in other cells treated with O-GNR-PEG-DSPE [24]. Although, the nanoribbons are functionalized with PEG-DSPE, we have observed O-GNR-PEG-DSPE aggregates forming near the cell membrane of cells in this and previous studies [22–24]. We believe the O-GNR-PEG-DSPE aggregates at the cell membrane due to specific interactions with the cell membrane and associated cytoskeletal components. We have observed this aggregation occurring before cells take up the nanoparticles through macropinocytosis.

Systematic evaluation of hematological effects of nanoparticles is critical toward their development for biomedical applications. Recent studies show that O-GNR-PEG-DSPE can be potentially employed as agents for drug-delivery [22,23] and imaging [16,21]. However, interactions of nanoribbons with hematological components have not been reported in these studies. The above results taken together indicate that O-GNR-PEG-DSPE formulation in blood could be potential safe up to the 80 µg/ml concentration for these applications. However, it must be mentioned that our previous drug-delivery studies have shown that concentrations below 10 µg/ml O-GNR-PEG-DSPE are relatively less efficacious compared with higher concentrations. Although, the *in vitro* results in this article identify a range of concentrations that should not adversely affect the hematological components, additional *in vivo* hematological and vasoactivity studies are necessary to obtain a thorough and complete assessment of their effects on the cardiovascular system. To this end, these studies in rodents are currently underway.

## Conclusion & future perspective

O-GNR-PEG-DSPEs elicit low concentration-dependent deformation of RBCs which does not lead to hemolysis. Exposure of the nanoribbons up to 80 µg/ml (1 h) does not induce histamine release from mast cells, PF4 activation in platelets and complement activation. However, a small decrease (˜5–10%) in anti-inflammatory cytokine levels was observed at all dosages tested (20–80 µg/ml for 1 h). The nanoribbons show significant uptake into endothelial cells and exhibit a concentration dependent decrease in endothelial cell viability. Taken together the results indicate that the exposure of O-GNR-PEG-DSPE formulation is potentially safe to the hematological components up to 80 µg/ml concentrations. These results lay the foundation for the use of these nanoribbons at potentially safe doses as *in vitro* and *in vivo* investigations/applications of the circulatory system.

**Table 1.** **Characterization of O-GNR-PEG-DSPE.**

**Nanoparticle**	**O-GNR-PEG-DSPE**
Dimensions	60–90 (w) × 500–1500(l)
Raman peaks	1340 cm^-1^(D) 1580 (G)
I_D_/I_G_	1.28
ζ potential	-26.30
Hydrodynamic diameter	457.5 ±35.70
TGA	10% weight loss (0–100°C) 30% weight loss (100–200°C) 25% weight loss (>200°C)
FTIR troughs	1070 cm^-1^1400 cm^-1^1605 cm^-1^ 1732 cm^-1^

FTIR: Fourier trandsform infrared spectroscopy; O-GNR: Ozed graphene nanoribbon; PEG-DSPE: 1, 2-distearoyl-*sn*-glycero-3-phosphoethanolamine-*N* (amino [polyethylene glycol]); TGA: Thermogravimetric analysis.

**Executive summary**

This is the first study that evaluates the potential interaction of graphene nanoribbons with key components of the circulatory system when injected in vivo for biomedical applications.
The study shows that functionalizing graphene nanoribbons with PEG-DSPE (1, 2-distearoyl-*sn*-glycero-3-phosphoethanolamine-*N* [amino (polyethylene glycol)]) decreases protein binding to the nanoparticles and prevents RBC hemolysis or activation of allergen, immune activation or blood clotting cascade.
Graphene nanoribbons (O-GNR)-PEG-DSPEs can potentially interact and cause structural changes in a small fraction of exposed RBCs.
O-GNR-PEG-DSPEs show a concentration dependent decrease in cell viability when exposed to endothelial cells with approximately 15% decrease at 100 µg/ml and approximately 40% decrease at 600 µg/ml exposure concentrations.
The toxicity observed is probably due to high cellular uptake of O-GNR-PEG-DSPEs.
O-GNR-PEG-DSPEs are safe for biomedical applications up to a concentration of 80 µg/ml in the blood
